# Acanthocytes and chorea: a diagnostic trap beyond neuroacanthocytosis

**DOI:** 10.1055/s-0046-1829048

**Published:** 2026-10-23

**Authors:** Layla Panini Laborda, Eder Alencar Moura, Lucas de Rosa Montanha, Sarah Nobre Quintão Brant, Igor Leonardo Vieira Caetano, Jacy Bezerra Parmera, Leandro Tavares Lucato, Fernando Freua

**Affiliations:** 1 Universidade de São Paulo, Faculdade de Medicina, Hospital das Clínicas, Departamento de Neurologia, São Paulo SP, Brazil.; 2 Universidade de São Paulo, Faculdade de Medicina, Hospital das Clínicas, Departamento de Hematologia, São Paulo SP, Brazil.; 3 Universidade de São Paulo, Faculdade de Medicina, Hospital das Clínicas, Instituto de Radiologia, São Paulo, SP, Brazil.

A 35-year-old woman, with history of psychiatric symptoms and spasms, progressed over 6 years to choreatic movements, frequent falls, and gait incoordination. Within a year, she became bedridden with severe dysarthria, generalized spasticity, hand dystonia, and pyramidal signs. 1 2 Blood tests revealed 17% acanthocytes ( Figures. 1 2 ), initially suggesting neuroacanthocytosis. Creatine kinase levels were within normal limits. Brain magnetic resonance imaging (MRI) was performed ( Figure 3 ), and whole exome sequencing with ExpansionHunter (Illumina Inc.) analysis confirmed Huntington's disease (49 CAG repeats in the *HTT* gene). 1 This case reinforces a previously described 3 4 but underrecognized association and highlights potential diagnostic pitfalls in patients presenting with chorea and acanthocytosis.

**Figure 1:**
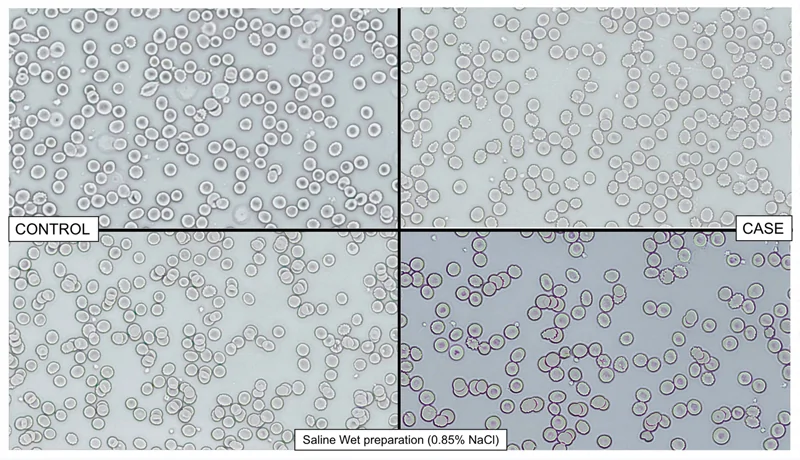
Peripheral blood smear analyzed in the central region of the slide after dilution in 0.85% saline, according to the protocol adapted from Feinberg (1991) and Storch (2005), compared with a healthy control. Red blood cells with morphology consistent with acanthocytes are observed.

**Figure 3:**
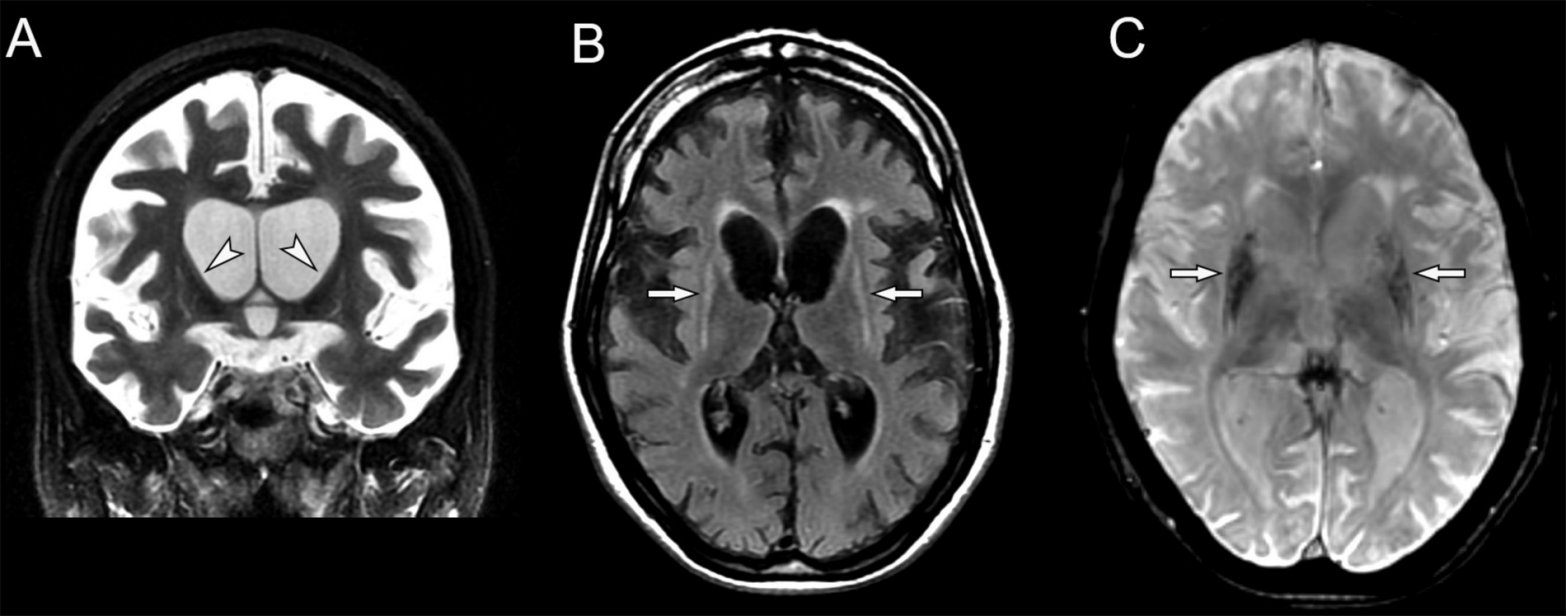
Brain magnetic resonance imaging (MRI). Coronal T2-weighted ( **A** ), axial fluid-attenuated inversion recovery (FLAIR) ( **B** ) and axial susceptibility-weighted ( **C** ) images demonstrate a clear reduction in brain volume, unexpected for age. One can also appreciate relevant atrophy of the head of both caudate nuclei (arrowheads in A). There is T2/FLAIR hyperintensity of the lentiform nuclei (arrows in **B** ), together with abnormal iron deposition for age, seen as hypointensity in susceptibility-weighted image (arrows in **C** ), signaling some degree of neurodegeneration.

**Figure 2:**
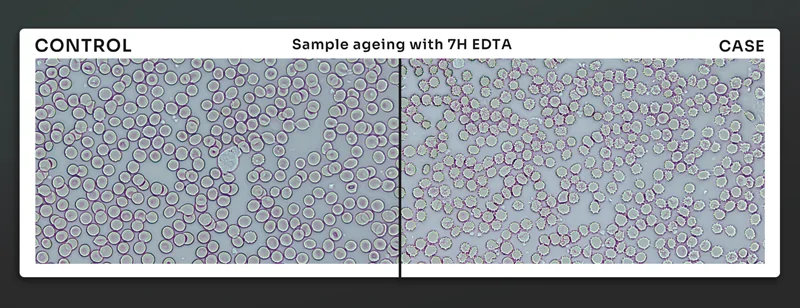
Smear image of the same sample after 7 hours of aging in EDTA, showing > 90% acanthocytes in the case compared with < 50% in the control.
